# Supplementary material for: Rat superior colliculus encodes the transition between static and dynamic vision modes
Source: Nat Commun. 2024 Feb 12;15:849. doi: 10.1038/s41467-024-44934-8 (PMC10861507; doi:10.1038/s41467-024-44934-8)
Supplement: Supplementary file 1 — Supplementary Information [file 41467_2024_44934_MOESM1_ESM.pdf]

Supplementary Information

Rat superior colliculus encodes the transition between static and dynamic vision modes

Rita Gil*, Mafalda Valente*, Noam Shemesh†

Champalimaud Research, Champalimaud Foundation, Lisbon Portugal

One Sentence Summary: The rat superior colliculus plays a critical role in discriminating temporal frequency.

†Correspondence:

Dr. Noam Shemesh

Champalimaud Research, Champalimaud Centre for the Unknown

Av. Brasilia 1400-038, Lisbon, Portugal.

E-mail: noam.shemesh@neuro.fchampalimaud.org

Phone number: +351 210 480 000 ext. #4467.

The PDF file includes:

- Supplementary Results and Figures
    - Behaviour
        - Behaviour Discussion
        - Impact of dark adaption period on calculated FFF thresholds
    - Functional MRI
        - Oxygenation Percentage influence on fMRI responses
        - Spin Echo vs. Gradient Echo fMRI acquisitions
        - Stimulation Parameters influence on measured fMRI responses
        - Average runs for different stimulation frequencies
        - Time Profiles for ROIS outside of the visual pathway
        - fMRI Raw Data
    - Electrophysiology
        - Zoomed in LFP signal amplitudes
        - Spectrogram for remaining tested frequencies
        - fMRI and electrophysiology signals for a larger frequency space
        - LFP power and fMRI percent signal change at Steady-state" Correlation
        - LFP and MUA Convolution with HRF
        - Ibotenic acid lesions
- Supplementary References

Supplementary Results and Figures

Behaviour

Behaviour Discussion

Most studies investigating the continuity illusion have used a GO/NOGO task paradigm[1–3] where animals were trained to respond in one port in order to report one of two conditions, flickering or continuous stimulus, and to withhold any response in the case of the opposite condition. In the present task we went one step further and allowed animals to freely choose between two ports where each port corresponded to one light condition.

In the context of continuity illusion studies, behavioural tasks have been designed and constrained to approximate measures to FFF thresholds; however, to completely rule out the possibility of animals simply comparing low vs high frequencies instead of flicker vs continuous light would require complementary and more complex behavioural experiments than employed so far. Since results of such experiments may also depend on task design[2], we focused on ensuring that our task would (i) avoid biasing the animals towards one side port; (ii) ensure that 50% of the trials would deliver continuous light; (iii) reward "probe trials" in the flicker port to prevent the low vs high frequency possible comparison from taking place; and (iv) limit the presentation of frequencies above 8 Hz ("probe trials") to only 10% of the flicker trials so that animals would not perceive the reward in the continuous stimulus as uncertain.

Despite these efforts, the observed behavioural percentage of reports to the flicker port for the continuous stimulus conditions reaches 10.7% instead of the expected values closer to 0%. This decrease in performance is not present for the easy flicker conditions (**Figure S1A**), where reports are above ~95%. The increase in "flickering" reports for the continuous light condition could represent a limitation at two different levels: (1) a limitation in the animals' ability to discriminate the "true continuous"; or (2) a limitation in the design of the task. We theorise that the observed increase in "flickering" reports for the continuous light might be due to a limitation in the design of the task and not animal related. In line with this, during the initial phase of training, when animals are presented with only 2 Hz and continuous light, they reach performance levels of

98% and 97%, respectively. If the animals' ability to discriminate "true continuous" light was indeed impaired then the percentage of "flickering" reports would already manifest itself in the initial training phase. This hints at the possibility that showing "probe trials" (that can potentially be perceived as continuous but were rewarded in the flicker port) for 10% of flickering trials might still have been excessive. Although experience is not expected to modulate activity at the level of the SC, this is not necessarily true for up-stream associative areas of the brain that may adapt to contingencies of the task and try to optimize the rat's strategy[4–7]. Therefore, after some time performing the task, animals may learn that, although the type of trials (flicker vs continuous) are balanced to both sides in terms of trial quantity and the "probe trials" being rare, when uncertain of the stimulus, it might appear more profitable to respond to the flicker port.

When calculating the FFF threshold proxies for such a complex behaviour, one could argue that other parameters, besides the animals' performance, might have been taken into consideration. In **Figure S1** we show percentage of aborted trials (**Figure S1B**) as well as movement (**Figure S1C**) and reaction times (**Figure S1D**) for the different presented frequencies of stimulation. Potentially supporting the measured FFF threshold proxy, animals show a slightly decreased percentage of aborted trials (by not waiting the required 1000 ms) in the vicinity of the calculated threshold (highlighted in red), which might be due to an increased uncertainty in those trials. However, it is unclear how other parameters could be easily included and we consider this to be out of the scope of this paper.

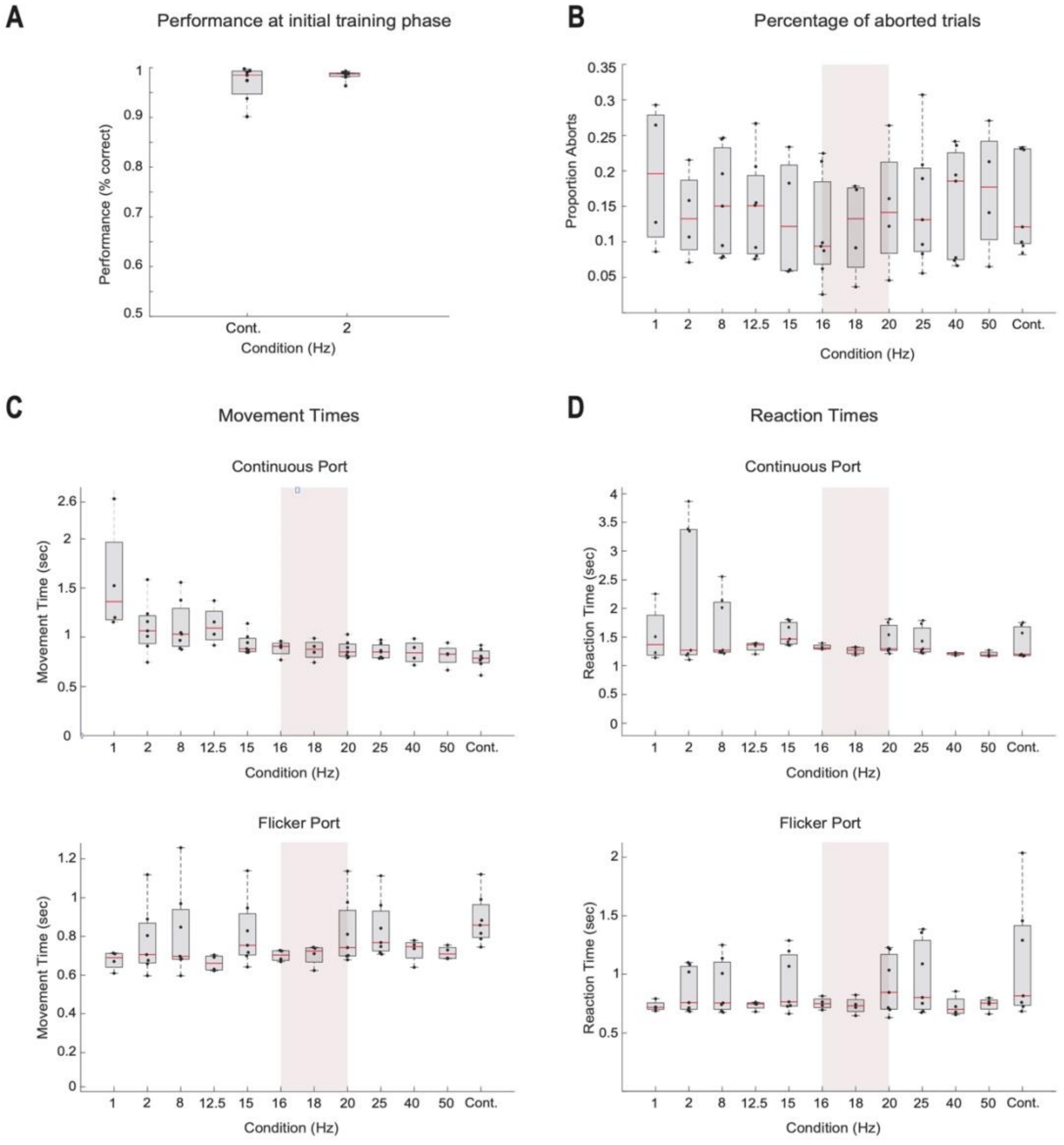

Figure S1: Behaviour Results (n=7). (A) Performance at initial training phase. Average performance of the animals for the initial training phase that included only the 2 Hz and "true" continuous stimuli. **(B) Percentage of aborted trials.** Percentage of trials aborted due to the animals attempting to respond before the mandatory minimum reaction time (1 s) reaching its end and the pure tone signalling the response period being played. **(C) Movement times**. Average over animals of the movement times - time it takes the animal to reach the response port once it leaves the central port after the 1 s minimum-reaction time has elapsed - registered for the different frequencies presented. These are organised according to the report port for the trial, continuous port (top) and flickering port (bottom). The red-shaded area marks the calculated FFF ± 2 Hz. **(D) Reaction times**. Average over animals of the reaction times - time the animal is exposed to the stimulus, 1 second mandatory time plus the time the animal choses to linger until ready to respond - registered for the different frequencies presented. These are organised according to the report port for the trial, continuous port (top) and flickering port (bottom). The red-shaded area marks the calculated FFF threshold: 18±2 Hz. Source data are provided as a Source Data file.

Impact of dark adaption period on calculated FFF thresholds

The impact of dark adaptation period on the obtained psychometric curve and calculated FFF threshold was investigated and is shown in **Figure S2**. Two curves were generated taking into consideration only trials that took place either 15 or 30 min, respectively, after the animals were placed inside the dark behavioural box. Similar thresholds were obtained for the two intervals (18±2 Hz and 17.8±2 Hz for the 15- and 30-min dark adaptation periods, respectively) highlighting animals sufficiently adapted their vision to the dark conditions after 15 minutes, at least for this particular task.

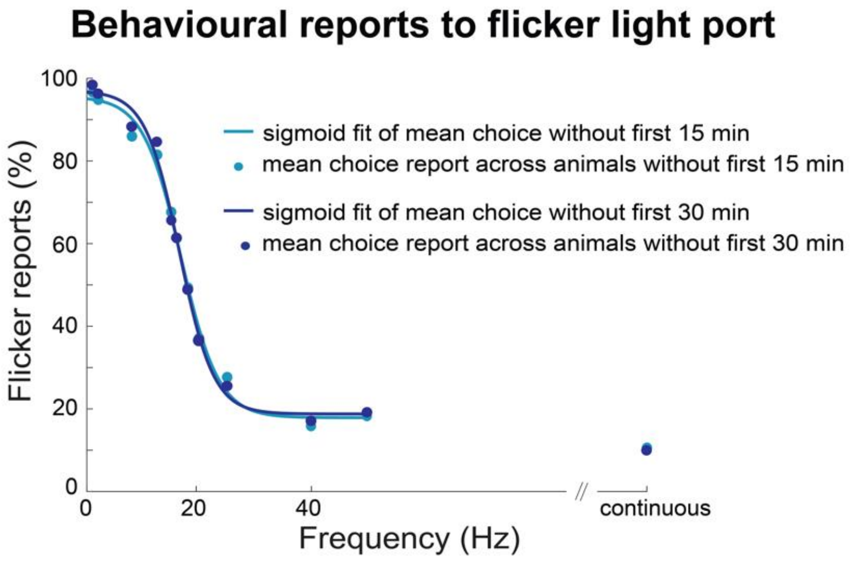

Figure S2: Psychometric curves obtained using only trials that took place after either 15 (light blue) or 30 (dark blue) min of dark adaptation. Similar results reveal that 15 min is already sufficient for the visual system of Long-Evans rats to adapt to the dark. The circles correspond to the averaged individual performances. Source data are provided as a Source Data file.

Functional MRI

Oxygenation Percentage influence on fMRI responses

Different oxygenation levels (medical air - 21% O_2; oxygen enriched air - 28% O_2; and hyperoxia - 95% O_2) were tested for two stimulation frequencies (2 Hz and 15 Hz) to investigate the sensitivity to measured positive and negative fMRI responses (**Figure S3**). Images were acquired with a SE-EPI sequence (TE/TR=43/1500 ms; FOV=18x16.1 mm; FA= 62°, resolution

= 269x268 μm^2; slice thickness=1.5 mm). Results reveal that, for both frequency regimes, the hyperoxia oxygenation state (95% O_2) is the best regime to maximize fMRI responses, both positive and negative, along the rat visual pathway.

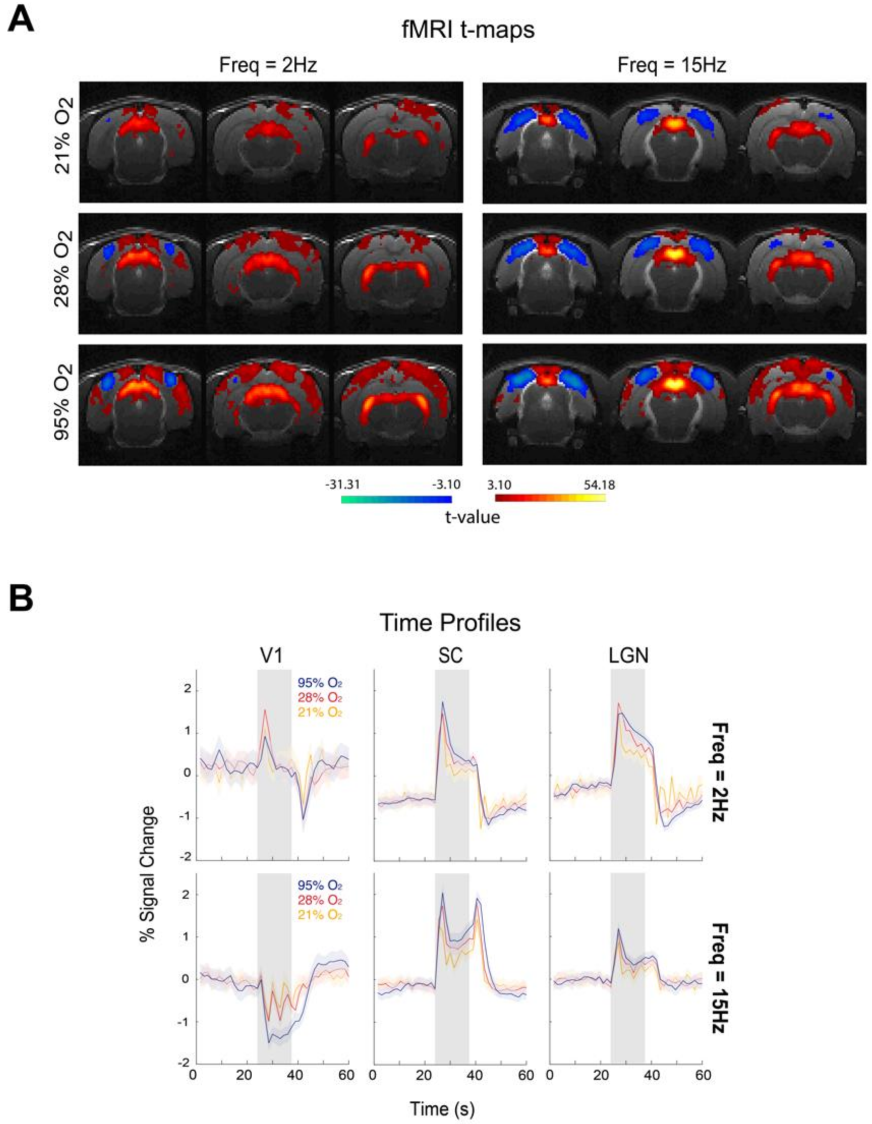

Figure S3: fMRI signal modulation with different percentages of oxygen. (A) fMRI t-maps for 21, 28 and 95% O_2 (p-value=0.001, cluster size=20 voxels). Bilateral fMRI responses along the main structures of the visual pathway are observed for both stimulation frequencies. Negative cortical fMRI responses along with positive subcortical fMRI responses are emphasised as the percentage of oxygen increases. For the 95% O_2 regime (hyperoxia) negative fMRI responses are observed in the V1B sub-regions (the binocular sub-region of the primary visual cortex) surrounded by positive responses in the rest of the primary visual cortex and secondary visual cortices. (**B) fMRI temporal profiles** for the different structures. An amplification of both positive and negative fMRI responses is confirmed for the hyperoxia condition. The amplification is mostly observed in cortical regions and more modest in subcortical regions. Legend: 21% O_2 (medical air)– yellow; 28% O_2 (oxygen enriched air) – orange; 95% O_2 (hyperoxia) – blue. Source data are provided as a Source Data file.

Spin Echo vs. Gradient Echo fMRI acquisitions

To investigate if fMRI signal modulations observed at different stimulation frequency were specific to the chosen sequence, spin echo (SE) EPI, we tested several stimulation conditions with a gradient echo (GE) EPI sequence with similar parameters (TE/TR=15/1500 ms; FOV=18x16.1 mm; FA= 62°, resolution = 269x268 μm^2; slice thickness=1.5 mm) as in the SE-EPI sequence.

Results shown in **Figure S4** reveal similar signal modulations observed in GE-EPI fMRI as the ones observed in SE-EPI acquisitions. Negative cortical responses appear first at 15 Hz stimulation frequency however, these appear more modest than the ones observed with SE-EPI acquisitions due to GE-EPI specific artefacts in these regions. SC responses show similar shifts from positive to negative responses as the stimulation frequency increases (at 25 Hz strong negative SC responses are observed). Due to enhanced specificity, the SE-EPI sequence was chosen for this study.

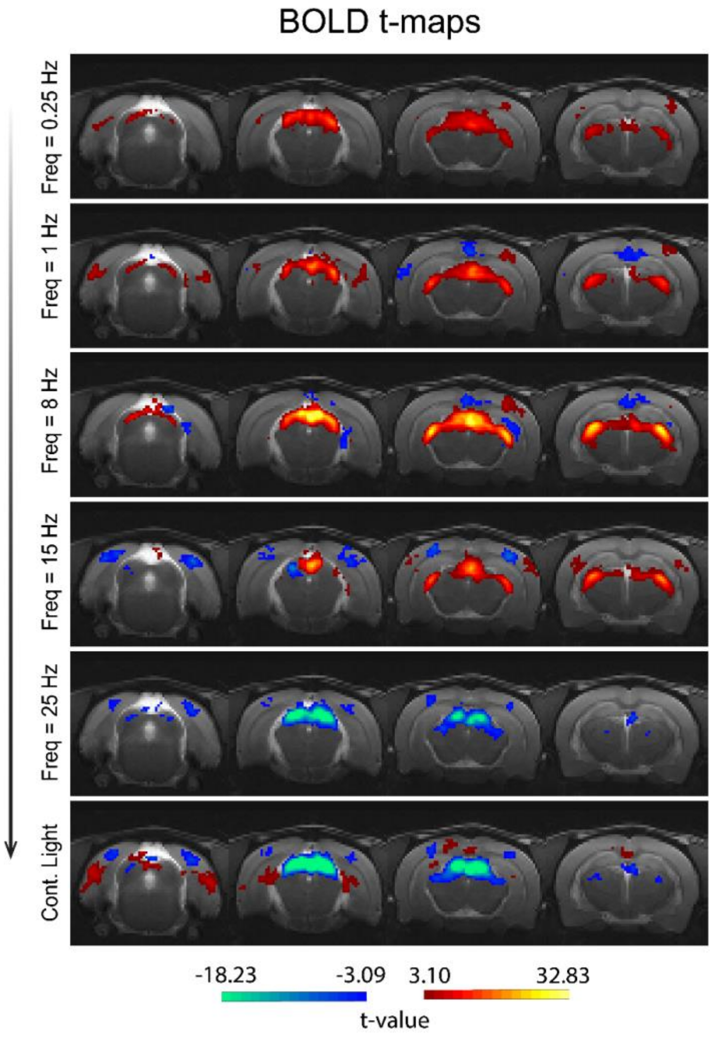

Figure S4: GE-EPI fMRI t-maps along different stimulation frequencies (cluster FDR corrected, p-value=0.001, cluster size=20 voxels). Bilateral fMRI responses are observed at the main visual pathway structures: SC, LGN and VC. Positive cortical responses at low stimulation frequencies are barely noticeable while stronger negative responses at frequencies higher than 15 Hz can be observed as in SE-EPI acquisitions. Subcortical responses also present signal modulations with strong negative responses in SC for frequencies higher than 25 Hz and reduced. LGN responses also appear modulated.

Stimulation Parameters influence on measured fMRI responses

From previous results we know that different frequencies with a constant flash duration of 10 ms (and consequently different inter-stimulus intervals - ISIs) induce fMRI signal modulations. To further investigate the stimulation parameter (ISI vs flash duration) that has major influence in such signal modulations we tested fMRI signal modulations keeping the same ISIs but modulating the flash duration from 10 ms to 1 s (**Figure S5**). These acquisitions were performed using the chosen SE-EPI sequence: TE/TR=40/1500ms; FOV=18x16.1mm; resolution = 269x268μm^2; slice thickness=1.5mm.

Results show that different flash durations originate similar results revealing that the change in ISIs is the main factor inducing fMRI signal modulations.

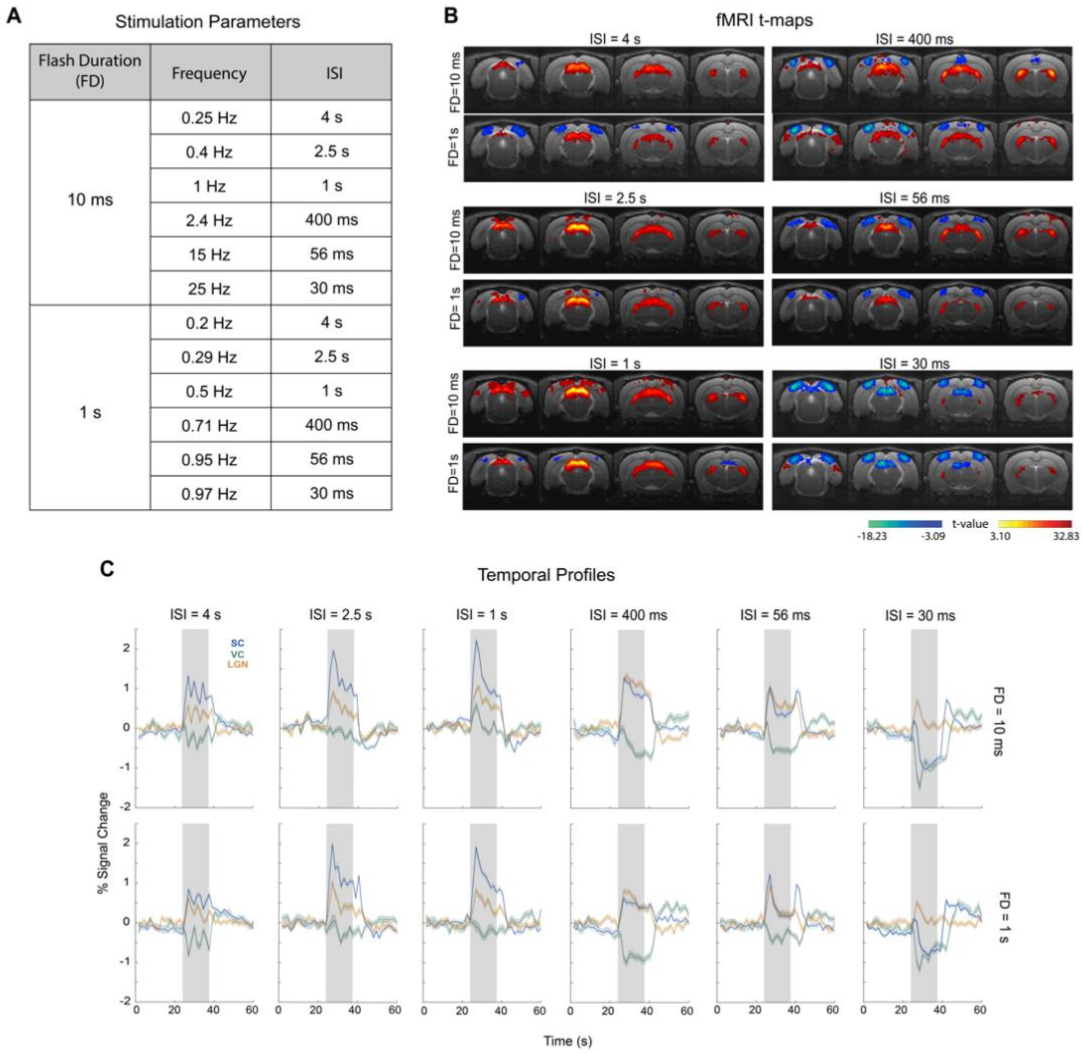

Flash Duration (FD)	Frequency	ISI
10 ms	0.25 Hz	4 s
	0.4 Hz	2.5 s
	1 Hz	1 s
	2.4 Hz	400 ms
	15 Hz	56 ms
	25 Hz	30 ms
1 s	0.2 Hz	4 s
	0.29 Hz	2.5 s
	0.5 Hz	1 s
	0.71 Hz	400 ms
	0.95 Hz	56 ms
	0.97 Hz	30 ms

Figure S5: Effects of stimulation parameters on fMRI response modulations along the visual pathway. (A) Stimulation Parameters for the different tested conditions. Two flash durations (FD) were

tested, 10 ms and 1 s, while keeping similar ISIs. **(B) fMRI t-maps** (p-value=0.001, cluster size=8 voxels, FDR cluster correction). Maps appear similar for both flash durations along the tested ISIs with increased cortical and subcortical negative fMRI signals at high frequencies; **(C) fMRI Temporal Profiles.** To further investigate how stimulation parameters affected fMRI signal modulations we plot the temporal profiles of signals with different FDs. These plots show similar curves for the three different ROIs (superior colliculus, SC, lateral geniculate thalamic nucleus, LGN, and visual cortex, VC) for the two different FDs. Modulations appear similar as the ISIs decrease. These results reveal that the ISI is the major factor inducing such changes. Source data are provided as a Source Data file.

<u>Average run for different stimulation frequencies</u>

Average runs from the chosen SE-EPI sequence are shown in **Figure S6** for the different drawn ROIs along the visual pathway and different stimulation frequencies.

Responses are consistent along runs and no habituation is observable along the cycles for any visual pathway structure.

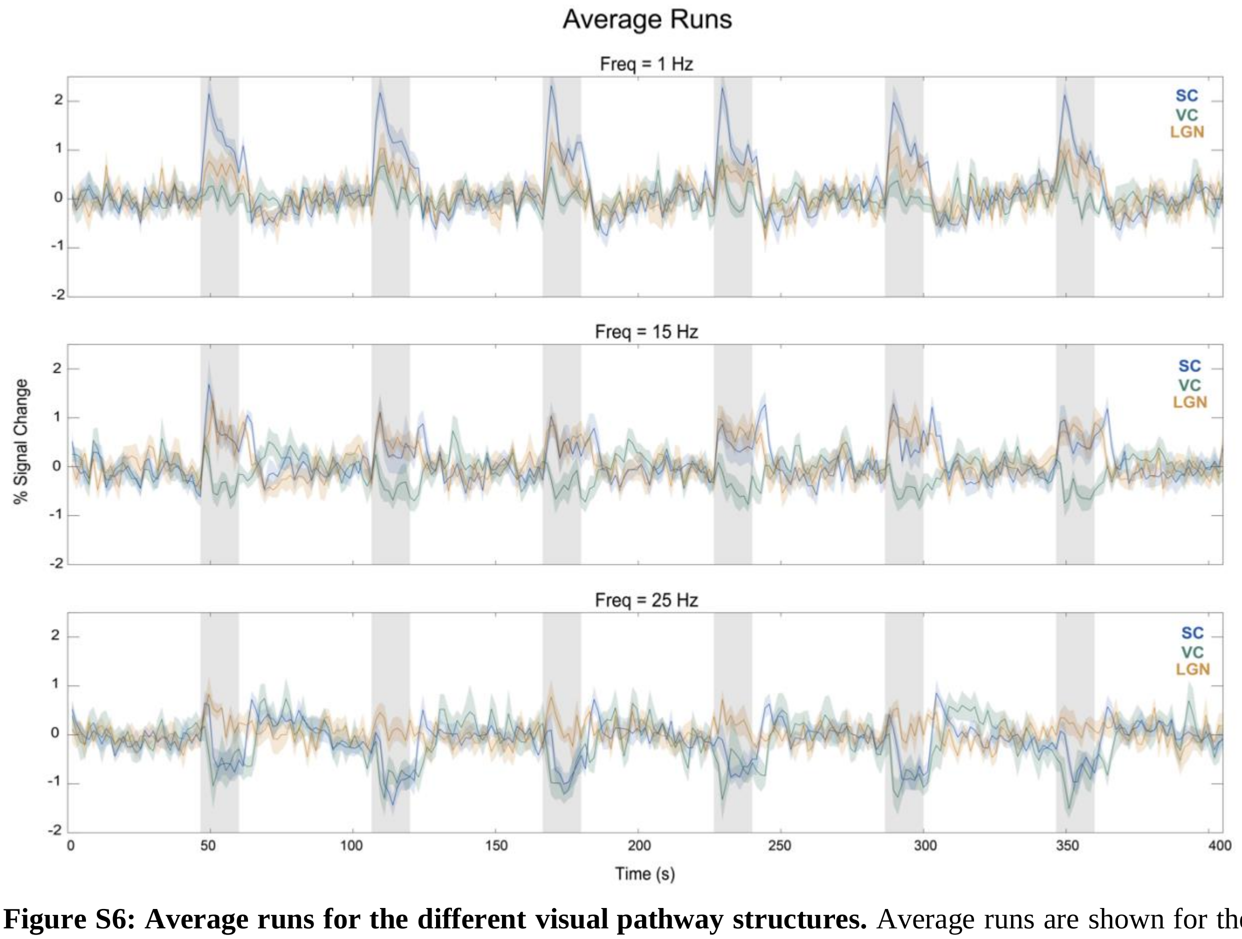

Figure S6: Average runs for the different visual pathway structures. Average runs are shown for the three ROIs – VC (green), SC (blue) and LGN (orange) – for three different stimulation frequencies – 1 Hz, 15 Hz and 25 Hz. Results show robust fMRI signal changes upon visual stimulation and no evidence of habituation along the stimulation cycles. Source data are provided as a Source Data file.

Time Profiles for ROIS outside of the visual pathway

Regions of interest outside of the visual pathway were drawn in order to investigate the involvement of other structures with temporal frequency discrimination. None of the investigated structures showed a meaningful signal response with the paradigm or with stimulation frequency (**Figure S7**):

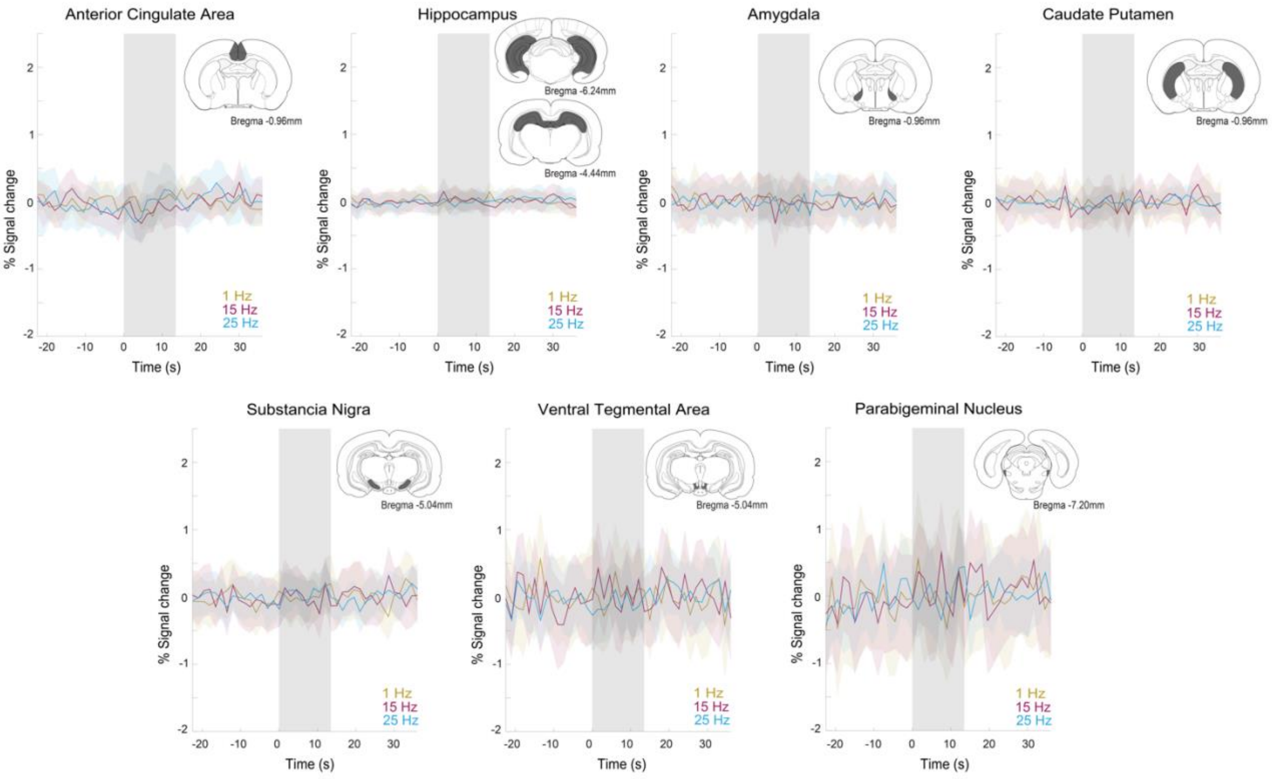

Figure S7: Time profiles for regions of interest outside the visual pathway. The involvement of other structures outside the visual pathway (caudate putamen, substantia nigra, ventral tegmental area, parabigeminal nucleus, amygdala, hippocampus, anterior cingulate area) with temporal frequency discrimination was investigated. From the obtained time profiles, no clear trend was observed with change of stimulation frequency for any of these regions. Source data are provided as a Source Data file.

fMRI Raw Data

Data was acquired using a 9.4T BioSpec scanner (Bruker, Karlsruhe, Germany) with an 86 mm quadrature resonator for transmittance and a 4-element array cryoprobe for signal reception. A SE-EPI sequence was used: TE/TR=40/1500 ms, partial Fourier coefficient=1.5, FOV=18x16.1 mm^2, resolution=269x268 μm^2, slice thickness=1.5 mm, t_{acq}=7 min 30 s.

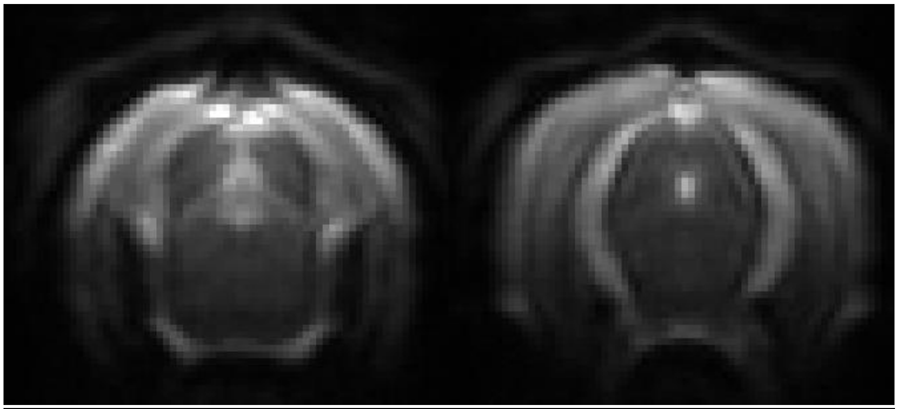

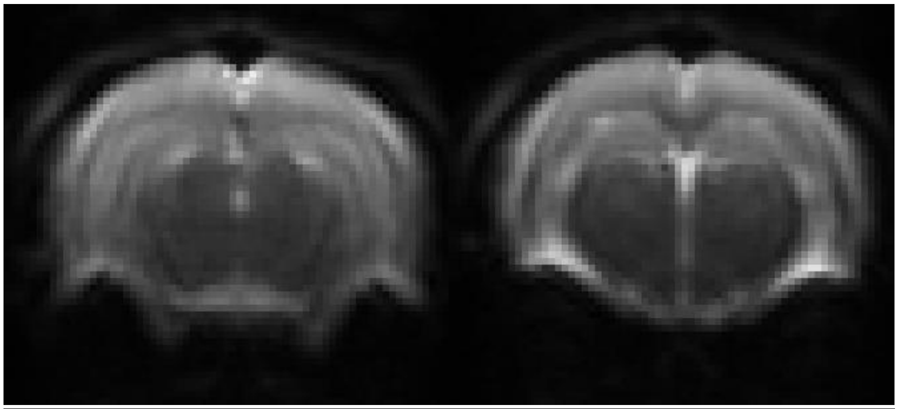

Electrophysiology

Zoomed in LFP signal amplitudes

Median LFP signal fluctuations reveal for the 1 Hz stimulation condition strong LFP oscillations induced by each flash. Closer inspection of the time profiles around the beginning and end of the stimulation period (**Figure S8**) reveals, similarly to the 1 Hz condition, that a single flash induces LFPs during the 15 Hz and 25 Hz stimulation period, albeit with smaller amplitude as the frequency increases. This feature is completely lost in the true continuous light stimulation condition. One similarity between the three conditions is the LFP onset oscillation while the offset oscillation is only present for the highest frequencies.

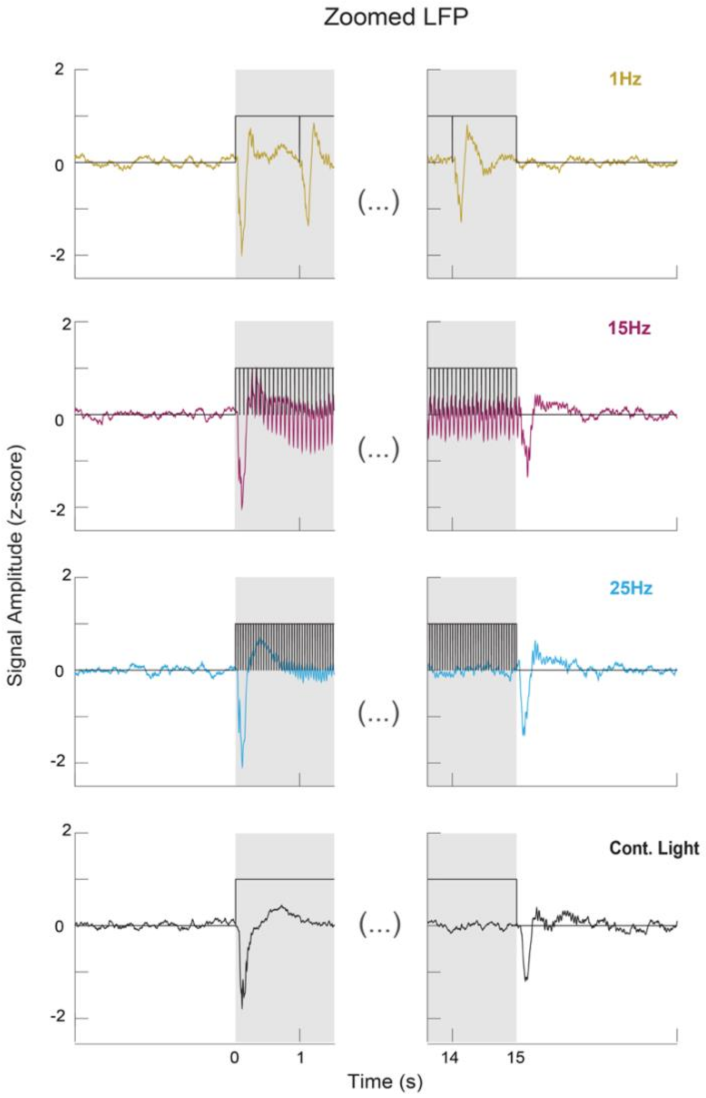

Figure S8: Zoomed mean LFP traces. Animal-averaged zoomed traces for the beginning and end of stimulation for the 1, 15, 25 Hz and continuous light stimulation conditions. Black lines represent individual

flashes. These plots confirm the individual flash induced oscillations and the absence of offset oscillations for the 1 Hz condition. For the 15Hz and 25 Hz conditions, smaller individual flash-induced oscillations are present along with marked onset and offset oscillations. For the continuous light condition only onset and offset oscillation are observed. Source data are provided as a Source Data file.

Spectrogram for remaining tested frequencies

In **Figure S9** we show, similarly to what is shown in **Figure 3C**, spectrograms between 0-50 Hz for the remaining tested frequencies.

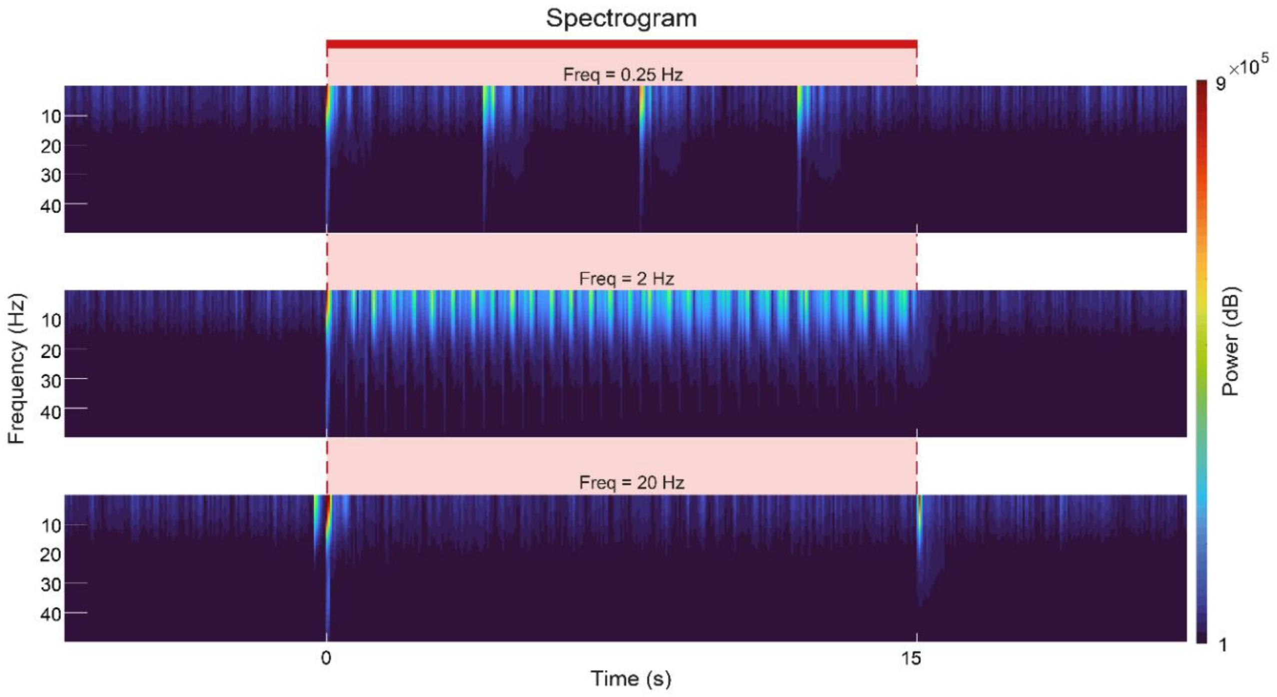

Figure S9: Spectrograms from electrophysiological recordings. (A) Spectrograms between 1-50 Hz. These plots confirm the individual flash induced power increases and the absence of offset oscillations for the 0.25 Hz and 2 Hz condition. For the 20 Hz condition onset and offset power increases are observed similarly to the 25 Hz;

fMRI and electrophysiology signals for a larger frequency space

To better characterise the evolution of the electrophysiological signal along different frequencies a wider range of frequencies was tested and the extra frequencies are shown in **Figure S10**.

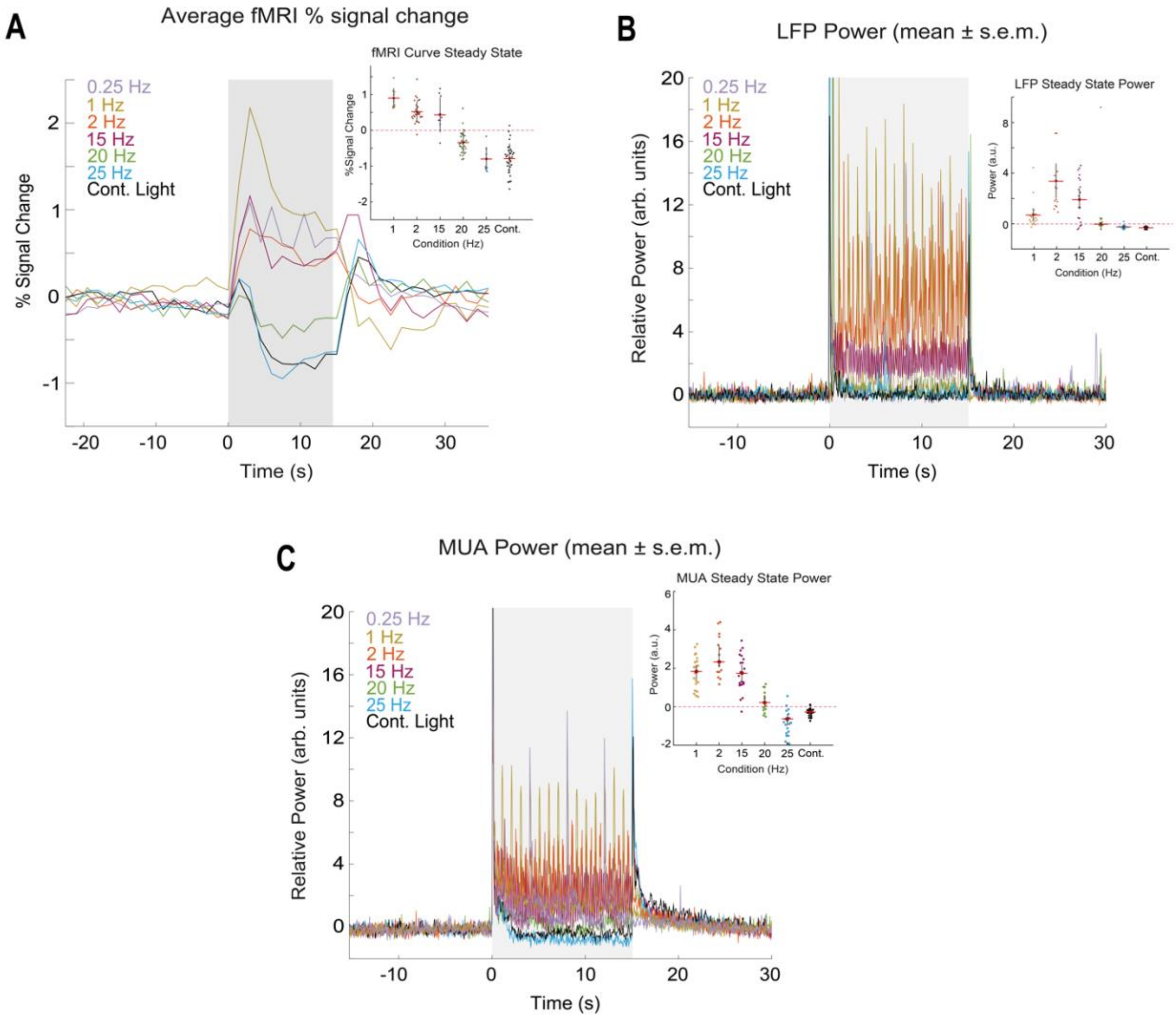

Figure S10: (A) fMRI time profiles. Higher stimulation frequencies lead to stronger SC NBRs; **(B) LFP relative power.** LFP power for all tested frequencies where stronger power reduction is observed for the 20, 25Hz and continuous light conditions; **(C) MUA relative power.** Similar trends as the ones observed for the LFP band. Interestingly high frequencies induced even a stronger MUA power reduction below baseline levels. Source data are provided as a Source Data file.

<u>LFP power plots and SC fMRI percent signal change at Steady-state” Correlations</u>

Figure S11 shows correlations between LFP signals and SC fMRI signals during “steady-state”.

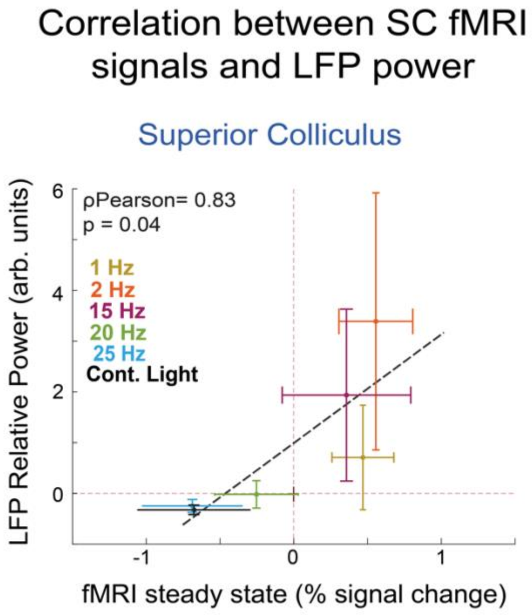

Figure S11: Correlation between SC fMRI signals and LFP power at steady-state. The correlation between SC and LFP power failed to be statistically significant with a coefficient of $\rho_{Pearson}(4) = 0.83$ (P = 0.004, 95% CI = [0.05, 0.98], two-tailed Pearson's). The circles represent the mean LFP power and fMRI percent signal change across animals while the error bars represent the standard deviation of the mean LFP power and fMRI percent signal change across runs. Source data are provided as a Source Data file.

LFP and MUA Convolution with HRF

In **Figure S12** the convolution of a typical HRF (peaking at 1 s) with electrophysiological power plots was performed in order to observe how such convolved signals would resemble fast fMRI responses. While onset and offset peaks between fast fMRI responses (n=6) and convolved signals appear well aligned, the amplitude of the negative fMRI responses observed at high stimulation frequencies cannot be completely explained. This means that other phenomena captured by the fMRI signals and not by the electrophysiological ones emphasise the negative amplitudes.

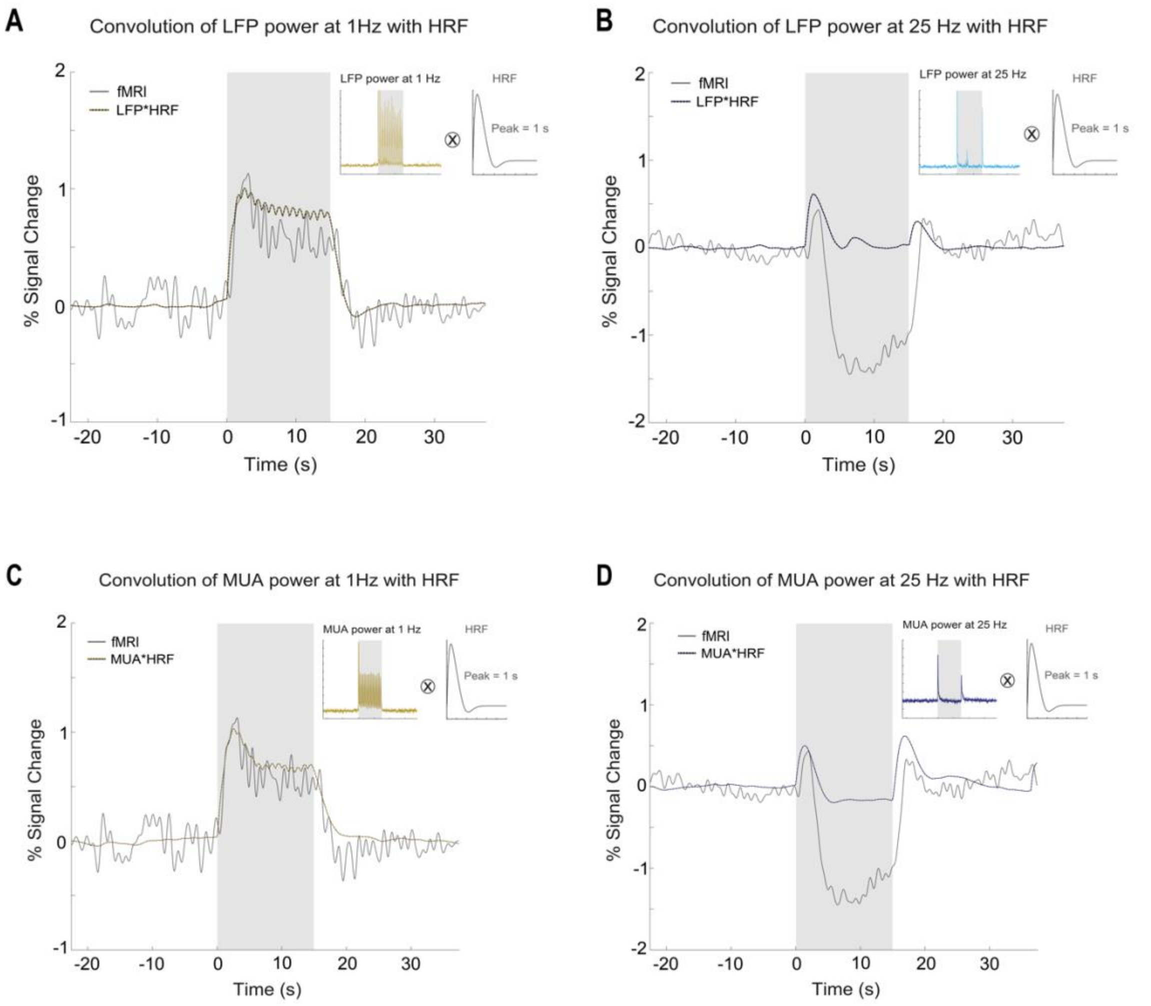

Figure S12: Convolution of electrophysiological data with an HRF peaking at 1 s. LFP convolutions for the 1 Hz (A) and 25 Hz (B) condition. The resulting convolved LFPs were compared with a fast fMRI acquisition (TR = 500 ms). Onset/offset peaks between the two curves appear aligned. **MUA convolution for the 1 Hz (C) and 25 Hz (D) condition.** The resulting convolved MUA was compared with a fast fMRI acquisition (TR = 500 ms). Onset/offset peaks between the two curves are aligned with onsets occurring ~1.5-2sec after stimulation started and offsets peaking ~1.8-2.3 sec after stimulation ended. Source data are provided as a Source Data file.

<u>Ibotenic acid lesions</u>

Figure S13 depicts further results for the ibotenic acid lesions in V1 (n=10). As expected, V1 temporal profiles appear flat with no positive to negative fMRI signal shifts with increasing stimulation frequency as was observed for the control regime. Temporal profiles in LGN are very similar between the lesion and control group, suggesting that the V1 lesion does not strongly affect processing in LGN. Lesions were separated from the fMRI experiments by one week to avoid inflammation and swelling artefacts in the images.

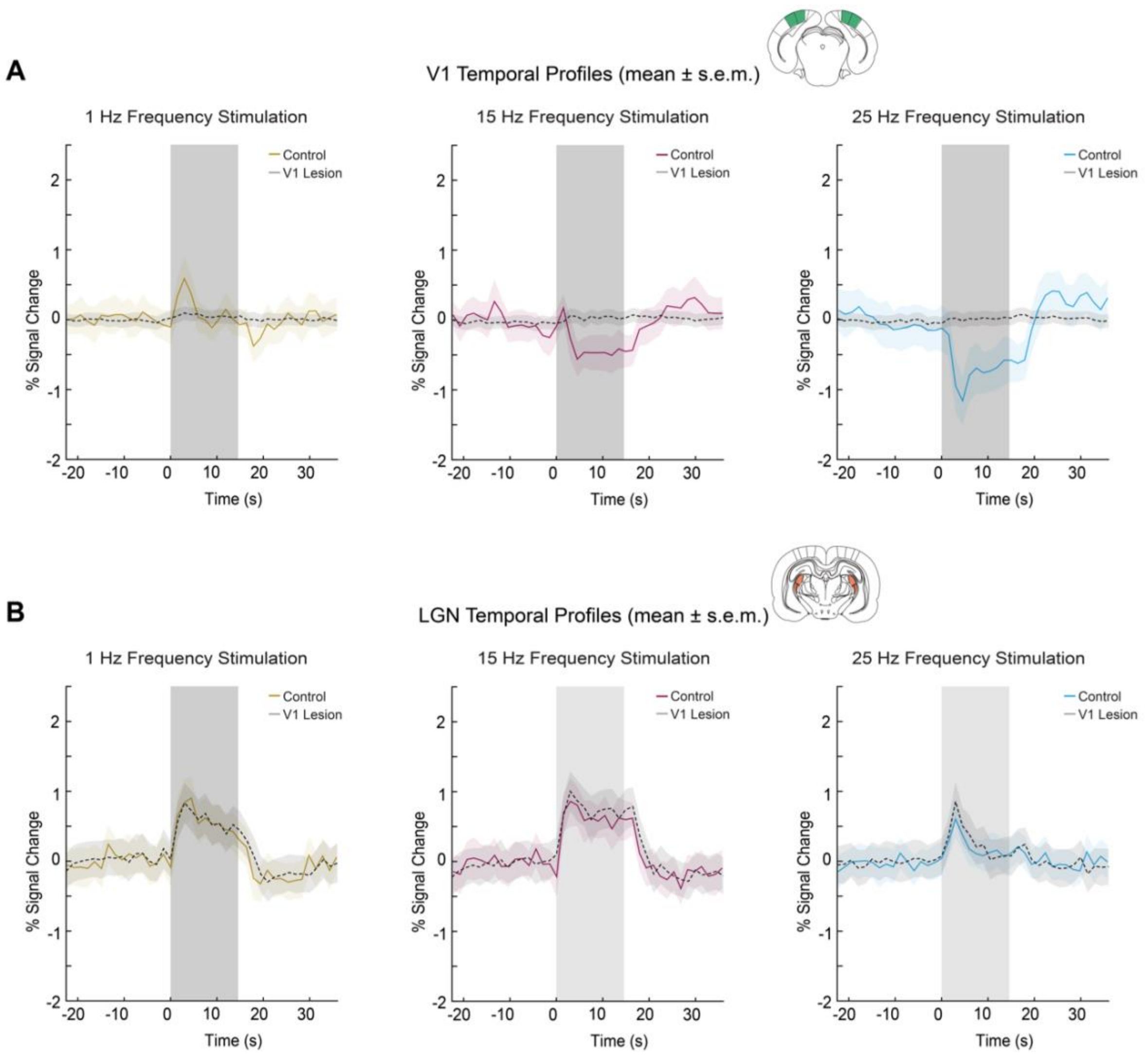

Figure S13: Cortical and thalamic fMRI temporal profiles after V1 ibotenic lesion (mean ± s.e.m. across animals). V1 profiles **(A)** appear flat as expected from localised ibotenic acid lesions while LGN profiles **(B)** appear similar to the control conditions with clear modulation as stimulation frequency increases but never reaching negative values. Source data are provided as a Source Data file.

Supplementary References

1. Shumake, S. A., Smith, J. C. & Taylor, H. L. Critical Fusion Frequency in Rhesus Monkeys. *Psychol Rec* **18**, 537–542 (1968).
2. Nomura, Y. *et al.* Evaluation of critical flicker-fusion frequency measurement methods using a touchscreen-based visual temporal discrimination task in the behaving mouse. *Neurosci Res* **148**, 28–33 (2019).
3. Ann Williams, R. *et al.* Flicker Detection in the Albino Rat Following Light-induced Retinal Damage. *Physiol Behav* **34**, 259–266 (1985).
4. Garvert, M. M., Moutoussis, M., Kurth-Nelson, Z., Behrens, T. E. J. & Dolan, R. J. Learning-Induced plasticity in medial prefrontal cortex predicts preference malleability. *Neuron* **85**, 418–428 (2015).
5. Herdener, M. *et al.* Musical training induces functional plasticity in human hippocampus. *Journal of Neuroscience* **30**, 1377–1384 (2010).
6. Yang, T. & Maunsell, J. H. R. The Effect of Perceptual Learning on Neuronal Responses in Monkey Visual Area V4. *Journal of Neuroscience* **24**, 1617–1626 (2004).
7. Lee, T. S., Yang, C. F., Romero, R. D. & Mumford, D. Neural activity in early visual cortex reflects behavioral experience and higher-order perceptual saliency. *Nat Neurosci* **5**, 589–597 (2002).
