# Supplementary material for: Rat superior colliculus encodes the transition between static and dynamic vision modes
Source: Nat Commun. 2024 Feb 12;15:849. doi: 10.1038/s41467-024-44934-8 (PMC10861507; doi:10.1038/s41467-024-44934-8)
Supplement: Supplementary file 3 — Description of Additional Supplementary Files [file 41467_2024_44934_MOESM3_ESM.pdf]

Description of Additional Supplementary Files:

Supplementary Movie 1: The file "SupplementaryMovie.mp4" depicts a representative part of the behaviour sessions. The animal starts new trials by poking on the central port and then choosing the side ports depending on the frequency displayed on the above head light (on the selected session the continuous and flickering lights would be rewarded in the right and left ports, respectively).
