# Supplementary material for: Rat superior colliculus encodes the transition between static and dynamic vision modes
Source: Nat Commun. 2024 Feb 12;15:849. doi: 10.1038/s41467-024-44934-8 (PMC10861507; doi:10.1038/s41467-024-44934-8)
Supplement: Supplementary file 5 — Reporting Summary [file 41467_2024_44934_MOESM5_ESM.pdf]

nature portfolio

Corresponding author(s): Noam Shemesh

Last updated by author(s): Dec 12, 2023

Reporting Summary

Nature Portfolio wishes to improve the reproducibility of the work that we publish. This form provides structure for consistency and transparency in reporting. For further information on Nature Portfolio policies, see our Editorial Policies and the Editorial Policy Checklist.

Statistics

For all statistical analyses, confirm that the following items are present in the figure legend, table legend, main text, or Methods section.

n/a	Confirmed	
☐	☒	The exact sample size (*n*) for each experimental group/condition, given as a discrete number and unit of measurement
☐	☒	A statement on whether measurements were taken from distinct samples or whether the same sample was measured repeatedly
☐	☒	The statistical test(s) used AND whether they are one- or two-sided<br>*Only common tests should be described solely by name; describe more complex techniques in the Methods section.*
☐	☒	A description of all covariates tested
☐	☒	A description of any assumptions or corrections, such as tests of normality and adjustment for multiple comparisons
☐	☒	A full description of the statistical parameters including central tendency (e.g. means) or other basic estimates (e.g. regression coefficient) AND variation (e.g. standard deviation) or associated estimates of uncertainty (e.g. confidence intervals)
☐	☒	For null hypothesis testing, the test statistic (e.g. F, t, r) with confidence intervals, effect sizes, degrees of freedom and P value noted<br>*Give P values as exact values whenever suitable.*
☒	☐	For Bayesian analysis, information on the choice of priors and Markov chain Monte Carlo settings
☒	☐	For hierarchical and complex designs, identification of the appropriate level for tests and full reporting of outcomes
☐	☒	Estimates of effect sizes (e.g. Cohen's d, Pearson's r), indicating how they were calculated

Our web collection on statistics for biologists contains articles on many of the points above.

Software and code

Policy information about availability of computer code

Data collection	MRI data was acquired using ParaVision 6.0.1. Electrophysiology data was acquired using the Open Ephys software (v0.5.5.1 to v0.6.4). Behavioural data was acquired using Matlab v2012b (with psychotoolbox-3).
Data analysis	All data was analysed with MATLAB (Natick, Massachusetts: The MathWorks Inc.) v2023b. All the MATLAB code used to generate the results in this study is available as a figshare repository (https://doi.org/10.6084/m9.figshare.24804948).

For manuscripts utilizing custom algorithms or software that are central to the research but not yet described in published literature, software must be made available to editors and reviewers. We strongly encourage code deposition in a community repository (e.g. GitHub). See the Nature Portfolio guidelines for submitting code & software for further information.

Data

Policy information about availability of data

All manuscripts must include a data availability statement. This statement should provide the following information, where applicable:

- Accession codes, unique identifiers, or web links for publicly available datasets
- A description of any restrictions on data availability
- For clinical datasets or third party data, please ensure that the statement adheres to our policy

The raw data that supports the findings described in the paper is available as a figshare repository (https://doi.org/10.6084/m9.figshare.24804948).

Research involving human participants, their data, or biological material

Policy information about studies with human participants or human data. See also policy information about sex, gender (identity/presentation), and sexual orientation and race, ethnicity and racism.

Reporting on sex and gender	n/a
Reporting on race, ethnicity, or other socially relevant groupings	n/a
Population characteristics	n/a
Recruitment	n/a
Ethics oversight	n/a

Note that full information on the approval of the study protocol must also be provided in the manuscript.

Field-specific reporting

Please select the one below that is the best fit for your research. If you are not sure, read the appropriate sections before making your selection.

[x] Life sciences [ ] Behavioural & social sciences [ ] Ecological, evolutionary & environmental sciences

For a reference copy of the document with all sections, see nature.com/documents/nr-reporting-summary-flat.pdf

Life sciences study design

All studies must disclose on these points even when the disclosure is negative.

Sample size	No statistical method was used to predetermine sample size. The sample sizes used in this work are similar or bigger than the ones used in previous studies using behaviour (Pardo-Vazquez, Jose L., et al., Nature neuroscience, 2019 and Khan, Adil Ghani, et al., Nature communications, 2012), fMRI (Schulz, Kristina, et al., Nature methods, 2012 and Rocchi, Federico, et al., Nature communications, 2022) and electrophysiology (Khan, Adil Ghani, et al., Nature communications, 2012 and Ito, Junji, et al., Nature communications, 2014) experiments.
Data exclusions	One animal was excluded from the behavioural task due to unreliable behaviour (>3sigma).
Replication	There was no replication of findings for each experiment modality. However the acquisitions (MRI, behaviour and electrophysiology) were performed in different batches of animals (males and females and different ages) and the results obtained were qualitatively similar to the population results.
Randomization	Allocation of animals to each experimental group was random.
Blinding	As controls are "within", blinding is irrelevant for this kind of study. Regarding the V1 lesion group, the lesion could be visible through the MRI images and therefore researchers knew the side of the lesion.

Reporting for specific materials, systems and methods

We require information from authors about some types of materials, experimental systems and methods used in many studies. Here, indicate whether each material, system or method listed is relevant to your study. If you are not sure if a list item applies to your research, read the appropriate section before selecting a response.

Materials & experimental systems

n/a	Involved in the study	
[x]	[ ]	Antibodies
[x]	[ ]	Eukaryotic cell lines
[x]	[ ]	Palaeontology and archaeology
[ ]	[x]	Animals and other organisms
[x]	[ ]	Clinical data
[x]	[ ]	Dual use research of concern
[x]	[ ]	Plants

Methods

n/a	Involved in the study	
[x]	[ ]	ChIP-seq
[x]	[ ]	Flow cytometry
[ ]	[x]	MRI-based neuroimaging

Animals and other research organisms

Policy information about studies involving animals; ARRIVE guidelines recommended for reporting animal research, and Sex and Gender in Research

Laboratory animals	This study used adult Long-Evans rats, both males and females aged between 8-29 week old.
Wild animals	All animals used were laboratory animals.
Reporting on sex	This study used both males and females without any specific criteria as this variable does not affect results.
Field-collected samples	The study did not involve samples collected from the field.
Ethics oversight	All animal care and experimental procedures were carried out according to the European Directive 2010/63 and pre-approved by the competent authorities, namely, the Champalimaud Animal Welfare Body and the Portuguese national veterinary agency (DGAV).

Note that full information on the approval of the study protocol must also be provided in the manuscript.

Magnetic resonance imaging

Experimental design

Design type	Block design
Design specifications	Stimulation Paradigm: 15 s stimulation followed by 45 s rest. Six blocks per run. Between 4-6 runs per session.
Behavioral performance measures	The animal's temperature was monitored during the entire experiment (36.5± 1oC) with an optic fibre rectal temperature probe (SA Instruments, Inc., Stony Brook, New York, USA) and was regulated using a heating system consisting of circulating water. Respiratory rate was measured using a pillow sensor (SA Instruments Inc., Stony Brook, USA). The animals had solely to see a visual stimulus that was placed in front of its eye so no other behavioural measured were needed besides making sure that the LED was delivering the light which was done via an arduino script.

Acquisition

Imaging type(s)	Functional and anatomical MRI
Field strength	9.4T
Sequence & imaging parameters	For correct slice placement along the visual pathway, an anatomical T2-weighted Rapid Acquisition with Refocused Echoes (RARE) sequence was used (TR/TE = 1600 / 36 ms, RARE factor = 8, Echo spacing = 9 ms; Averages = 3; FOV = 18 × 16. mm2, in-plane resolution = 168 × 150 µm2, slice thickness = 800 µm, tacq = 1 min 3 s). The functional MR imaging was acquired using a Spin-Echo Echo-Planar Imaging (SE-EPI) sequence (TE/TR = 40 / 1500 ms, PFT = 1.5, FOV = 18 x 16.1 mm2, in-plane resolution = 269 x 268 µm2, slice thickness = 1.5 mm, tacq= 6 min 50 s).
Area of acquisition	Eight slices were acquired in order to capture the entire visual pathway.

Diffusion MRI ☐ Used ☒ Not used

Preprocessing

Preprocessing software	SPM12 and Matlab v2016a were used during preprocessing. Pre-processing steps included manual outlier removal (a spline interpolation was made taking the entire time course), slice-timing correction (using a sinc-interpolation) followed by head motion correction (using mutual information). Data was afterwards co-registered to the T2-weighted anatomical images, normalised to a reference animal and smoothed using 3D Gaussian isotropic kernel with full width half-maximum corresponding to 1 voxel (0.268 mm).
Normalization	Data was normalized to a reference animal.
Normalization template	n/a
Noise and artifact removal	Motion correction was performed using a conventional mutual information algorithm.
Volume censoring	Outlier correction was performed, but fewer than 2% of the data were identified as outliers and corrected, not censored.

Statistical modeling & inference

Model type and settings	A general linear model (GLM) analysis was conducted where the stimulation paradigm was convolved with an hemodynamic response function peaking at 1 sec.
Effect(s) tested	ON/OFF

Specify type of analysis: ☐ Whole brain ☐ ROI-based ☒ Both

Anatomical location(s)	For the ROI analysis, the 6th Edition of Paxinos & Franklin's rat brain atlas served as guidance for the manual ROI delineation.
Statistic type for inference<br>(See Eklund et al. 2016)	A one-tailed voxelwise t-test was performed, tested for a minimum significance level of 0.001 with a minimum cluster size of 20 voxels.
Correction	Multiple comparison using a cluster false discovery rate test (FDR).

Models & analysis

n/a	Involved in the study
☒	☐ Functional and/or effective connectivity
☒	☐ Graph analysis
☒	☐ Multivariate modeling or predictive analysis
